# DNA Methylation Profile in Buffy Coat Identifies Methylation Differences Between Cirrhosis with and Without Hepatocellular Carcinoma

**DOI:** 10.3390/cancers17020266

**Published:** 2025-01-15

**Authors:** Hyeyeun Lim, Hashem B. El-Serag, Michelle Luster, Megan L. Grove, Jinyoung Byun, Yuri Jung, Younghun Han, Eric Boerwinkle, Christopher I. Amos, Aaron P. Thrift

**Affiliations:** 1 Section of Epidemiology and Population Sciences, Department of Medicine, Baylor College of Medicine, Houston, TX 77030, USA; u249494@bcm.edu; 2 Section of Gastroenterology and Hepatology, Department of Medicine, Baylor College of Medicine, Houston, TX 77030, USA; hasheme@bcm.edu (H.B.E.-S.); michelle.luster@bcm.edu (M.L.); 3 Human Genetics Center, Department of Epidemiology, School of Public Health, The University of Texas Health Science Center at Houston, Houston, TX 77030, USA; megan.l.grove@uth.tmc.edu (M.L.G.); eric.boerwinkle@uth.tmc.edu (E.B.); 4 Institute for Clinical and Translational Research, Baylor College of Medicine, Houston, TX 77030, USA; jinyoung.byun@bcm.edu (J.B.); younghun.han@bcm.edu (Y.H.); 5 Ridgewood High School, Ridgewood, NJ 07450, USA; yurijungrhs@gmail.com; 6 Dan L Duncan Comprehensive Cancer Center, Baylor College of Medicine, Houston, TX 77054, USA

**Keywords:** progression, methylation markers, *ADAM12*, *PSD3*, biomarkers, risk stratification, early detection, epigenetic changes, epigenome

## Abstract

Background/Objectives: Cirrhosis is the precursor to most cases of hepatocellular carcinoma (HCC). Understanding the mechanisms leading to the transition from cirrhosis to HCC and identifying key biomarkers is crucial to developing effective screening strategies and reducing HCC-related mortality. DNA methylation is associated with gene inactivation and plays an important role in physiological and pathological processes; however, its role in cirrhosis progression to HCC is unknown. Methods: We performed genome-wide DNA methylation profiling using Illumina Infinium MethylationEPI BeadChip in pre-diagnostic samples from 22 cirrhosis patients who subsequently developed HCC and 22 cirrhosis patients who remained HCC-free during an average 4-year follow-up. In a secondary analysis, we examined a subset of patients without hepatitis C virus (HCV) infection. Results: We identified three differentially methylated positions (DMPs) located in *ADAM12* (cg13674437) and *PSD3* (cg06758847 and cg24595678) that show a strong association with HCC risk (lower median vs. higher median hazards ratio (HR): HR _cg13674437_ = 0.34, 95% CI = 0.14–0.83; HR _cg06758847_ = 4.89, 95% CI = 1.79–13.33; HR _cg24595678_ = 11.19, 95% CI = 3.27–38.35). After excluding all HCV-active patients from our analysis, the HR for the DMPs remained significant. Conclusions: In conclusion, the findings in this study support the theory that buffy coat-derived DNA methylation markers could be used to identify biomarkers among cirrhosis patients at high risk for HCC before clinical symptoms appear. A further study with a large prospective cohort is required to validate these findings.

## Simple Summary

Cirrhosis is the precursor to most cases of hepatocellular carcinoma (HCC). HCC incidence and mortality have increased rapidly over the past two decades in the US, with a <20% 5-year survival rate. Understanding the mechanisms of this transition and identifying biomarkers is vital for effective screening and reducing HCC-related mortality. This study evaluated genome-wide DNA methylation patterns in the buffy coats of cirrhosis patients who developed HCC and those who remained HCC-free during an average four-year follow-up. We then identified differentially methylated sites, distinguishing cirrhosis with HCC from cirrhosis without HCC. The findings support the theory that buffy coat-derived DNA methylation markers could be implementable to stratify cirrhosis patients at high risk before clinical symptoms appear.

## 1. Introduction

Hepatocellular carcinoma (HCC) is a leading cause of death worldwide [1]. HCC incidence and mortality in the US have increased rapidly over the past two decades [2]. Despite advancements in treatment, a total cure remains possible for fewer than 10% of HCC patients, and the 5-year survival rate remains at <20% [3]. Cirrhosis is the precursor to more than 80% of HCCs diagnosed in the United States. Therefore, understanding the transition mechanism from cirrhosis to HCC and identifying key biomarkers are crucial steps to developing effective screening, risk stratification, and prevention strategies, and improving survival. 

DNA methylation is an epigenetic modification that can control gene expression and chromosomal stability without changing the DNA sequence [4]. Changes in methylation patterns have been observed in many solid cancers and play a key role in the biological mechanisms of carcinogenesis [5,6,7]. Previous studies that explored differentiated DNA methylation in tumor tissues have demonstrated changes in overall DNA methylation in the progression of liver disease from cirrhosis to early HCC [7,8,9,10,11]. These studies have also identified genes with differential methylation and expression levels and have suggested that differentially methylated CpG sites could be used to predict early-stage HCC [7,8]. An epigenomic study usedthe Cancer Genome Atlas (TCGA) datato profile HCC tissues compared to adjacent normal tissues. Then, they found that methylation levels of *CTF1*, *FZD8*, *PDK4*, and *ZNF334* were associated with worse overall survival [9]. A genome-wide DNA methylation study by Hernandez-Meza et al. found that tissue methylation levels in the promoter regions of *TSPYL5*, *KCNA3*, *LDBH*, and *SPINT2* increased from cirrhosis to early-stage HCC and were associated with lower gene expression levels [10]. Patients with metabolic dysfunction-associated steatotic liver disease (MASLD)-related HCC show higher methylation levels of *FLCN* and *WDR6* and lower methylation levels of *MAML3*, *TRIM4*, *PRC1*, *TUBA1B*, and *WHSC1* in tissue compared to patients with hepatitis B virus (HBV) or/and hepatitis C virus (HCV) infection and HCC [12,13]. An unsupervised hypermethylation clustering study identified that *PAX6* was more frequently hypermethylated in HCV-related HCC than in HBV-related HCC [14]. 

Multiple studies have shown cancer-specific aberrant DNA methylation in the blood of patients with breast, ovarian, colon, and prostate cancers. The studies suggested the application of blood, including buffy coats, for understanding the mechanism of cancer progression [15,16,17,18,19,20,21]. DNA methylation profiles in peripheral blood mononuclear cells (PBMCs), leukocytes, or white blood cells (WBCs) of patients with chronic HBV seemed different in those with liver cirrhosis or HCC. Differentially methylated promoters are associated with the progression of related liver disease and are linked to immune-related mechanisms [22,23,24]. Prospective studies have shown differentially methylated patterns in the blood (PBMCs or WBCs) between HCC patients and healthy individuals or cirrhosis patients and supported that these methylation changes before clinical symptoms appear are associated with the development of HCC [25,26,27]. However, these studies are limited to comparing HCC cases to healthy or hepatitis B virus-infected controls or exploring 450K CpG sites.

In this study, we performed genome-wide DNA methylation profiling in buffy coats for cirrhosis patients who developed HCC and cirrhosis patients who remained HCC-free during an average 4-year follow-up. We then identified differentially methylated sites using Illumina Infinium MethylationEPIC BeadChips array (Illumina, San Diego, CA, USA), which cover over 850 K CpG sites with better coverage of genomic regions. In addition, we evaluated the cell type-specific enrichment and pathway enrichment analyses to investigate the biological functions of the differentially methylated sites. 

## 2. Materials and Methods

### 2.1. Patient Recruitment and Sample Collection

In the current study, we used baseline pre-diagnostic samples from 22 cirrhosis patients who subsequently developed HCC (the HCC group) and baseline samples from 22 gender- and age-matched cirrhosis patients who remained cancer-free (the cirrhosis group) during an average 4-year follow-up (ranging from 1.4 to 5.9 years) from the Texas Hepatocellular Carcinoma Consortium (THCCC) cohort. We excluded samples from patients who were followed up for less than one year to limit potential time-related biases. The patients’ body mass index (BMI), age, race, smoking habits, diabetes, and pre-existing liver disease history were collected from electronic medical records. 

Patients with cirrhosis in the THCCC cohort were recruited from the Baylor St. Luke’s Medical Center and Michael E. DeBakey VA Medical Center in Houston between 4 January 2017 and 16 January 2019. Every 6-month follow-up was performed as part of clinical HCC surveillance. All participants provided written informed consent to participate. At the time of enrollment and blood collection, none of the participants had HCC. Whole blood samples were collected into EDTA tubes and underwent refrigerated centrifugation (4 °C 1300× *g*) for 10 mins to isolate buffy coat samples and then were subsequently shipped to the Population Sciences Biorepository Core at Baylor College of Medicine to extract DNA. This study was conducted in accordance with the Declaration of Helsinki, and the protocol was approved by the Institutional Review Board at Baylor College of Medicine. 

### 2.2. DNA Extraction and Bisulfite Conversion

DNA was extracted within five days of initial collection, and samples were frozen at −80 °C until analysis. DNA was extracted from the 400 µL buffy coats using the Qiagen Qiacube and a QIAamp DNA Blood Mini Qiacube Kit (cat no: 51126) according to the manufacturer’s instructions (Qiagen; Venlo, The Netherlands). DNA purity and concentration were estimated using a Quibt3.0 fluorometer (Thermo Fisher Scientific; Waltham, MA, USA). A total of 500 ng of DNA was treated with sodium bisulfite using an EZ-96 DNA Methylation Kit accruing to the manufacturer’s protocol (Zymo Research; Irvine, CA, USA) at the University of Texas Health Science Center at Houston, School of Public Health, Human Genetic Center Laboratory. 

### 2.3. DNA Methylation Quality Control and Data Processing

Genome-wide methylation analysis using Infinium MethylationEPIC BeadChips (Illumina, Inc.: San Diego, CA, USA) was performed according to the manufacturer’s protocol [28]. The BeadArray Controls Reporter tool (Illumina, Inc., San Diego, CA) was used to assess laboratory- or chemistry-related failures. All samples included in the analysis had a call rate > 99%. Genders were confirmed, and values clustered as expected after performing a principal component analysis using the wateRmelon R package (4.3.2) [28]. Median methylated/unmethylated (M/U) signals were reviewed, and no issues were detected. 

The Chip Analysis Methylation Pipeline (ChAMP) methylation analysis package was used in R (4.3.2) [29]. The probes that targeted the sex chromosomes, with <3 beads in at least 5% of the sample per probe, aligned to multiple locations, or those were associated with single nucleotide polymorphisms in the probe sequence were filtered for removal prior to analysis [30]. Beta values (β ranging from 0 to 1) were normalized using the beta mixture quantile dilation (BMIQ) algorithm to remove type 1 and type 2 probe bias [31]. The wateRmelon and ChAMP packages are available from Bioconductor (https://www.bioconductor.org, accessed on 1 October 2024). 

The normalized *β* values were corrected for the proportions of different cell types, except the lowest average cell type proportion. The FlowSorted.Blood.EPIC package of R (4.3.2) was used to estimate the cell type proportions of each of the major cell types present in the blood (B-cell, CD8+ T cell, CD4+ T cell, natural killer lymphocytes, monocytes, and neutrophils) [32]. We ran a Singular Value Decomposition (SVD) regression analysis to estimate the impact of the following covariates, array, plate, well, age, sex, diabetes, smoking, alcohol consumption, hypertension, BMI, and etiology of liver disease, on HCC prognosis and then adjusted for the covariates with *p*-values of <0.05 using ChAMP.runCombat function. CpG sides were annotated using EPICanno.ilm10b4.hg19. 

### 2.4. Differentially Methylated Positions (DMPs)

DMPs were identified as CpG sites with a Benjamini–Hochberg adjusted *p*-value (*q*-value) of <0.05 by using the ChAMP.DMP function which implements the limma package [33]. The DMPs were classified into their location relative to their genomic regions: promoter, 3′-UTR (3′untranslated region), gene body, exon boundary, and intergenic region. The promoter region was divided into 1st Exon, 5′-UTR (5′-untranslated region), TSS 1500 (within 1500 bp upstream of the transcription start site), and TSS200 (within 200 bp upstream of the transcription start site). The DMPs were also classified by distance from the CpG islands: CpG island, shore, shelf, and open sea. 

### 2.5. Gene Set Enrichment Analysis

To identify the biological functions and the signaling pathways involved in the genes corresponding to the DMPs, we performed the Kyoto Encyclopedia of Genes and Genomes (KEGG) pathway enrichment analysis using ShinyGO 0.8 (http://bioinformatics.sdstate.edu/go, accessed on 1 October 2024) [34,35]. False discovery rates (FDRs) were calculated using the Benjamini–Hochberg method. Fold enrichment was calculated by dividing the percentage of genes corresponding to the DMPs that belong to a pathway by the percentage of genes in the KEGG database that belong to that same pathway. 

### 2.6. Identification of DMPs to Distinguish Cirrhosis with HCC from Cirrhosis Without HCC

To identify the most important and specific DNA methylation sites for subjects who progressed to HCC, we restricted the analysis to DMPs with a *q*-value < 0.05 and an absolute β value difference of 0.15 or greater between cirrhosis and HCC. Using cirrhosis samples as a reference, we defined hyper-DMPs as DMPs with a β value difference equal to or greater than 0.15 and hypo-DMPs as those with a β value difference equal to or less than −0.15. 

We also identified highly associated DMPs with HCC prognosis based on the importance scores (>0.1) from a 5-fold cross-validation random forest (RF) analysis. RF is a machine-learning methodology that employs an ensemble of decision trees in which a random set of cases and controls, as well as a random set of features, is selected. It applies the optimal tree developed on a set of features and observations based on the observations that were not studied in a particular sample of the data. Each feature is ranked by ordering the importance score, which indicates how much of a decrease in prediction accuracy occurs when a variable is not included in a classification tree that is fitted as a part of the random forest analysis. The Caret R package v 6.0.94 was used to perform the RF model [36]. To rank the DMPs and covariates (age, sex, alcohol consumption, smoking behavior, hypertension, BMI, and cirrhosis etiology at baseline) according to how they affect the model predictions (importance score), we used the varImp function. 

In addition, we examined the association of the β values of the selected DMPs and covariates with the risk of HCC using univariate Cox proportional hazard regression, conducted using the survival R package v 3.7 [37]. The patients were grouped into two groups according to the β values for each DMP: high (the β value was higher than the median methylation level of the individual DMP) and low (the β value was lower than the median methylation level of the individual DMP). HCV infection is a well-known risk factor for inflammation in the liver, which can cause cirrhosis and HCC, as well as aberrant methylation patterns [38,39,40,41]. Thus, we conducted a secondary analysis stratified by HCV status at enrolment. 

## 3. Results

### 3.1. Patient and Tumor Characteristics

A total of 44 samples from 44 unique patients (22 in the HCC group and 22 in the cirrhosis group) were used for DNA methylation profiling. Of these patients, 72% were male, with a median age of 64 years (range 48–72 years) at baseline. HCV was the underlying etiology for over half (54.5%) of patients. The characteristics of the patients are shown in Table 1.

### 3.2. Genome-Wide DNA Methylation Landscape of HCC in Buffy Coats

**Figure 1:**
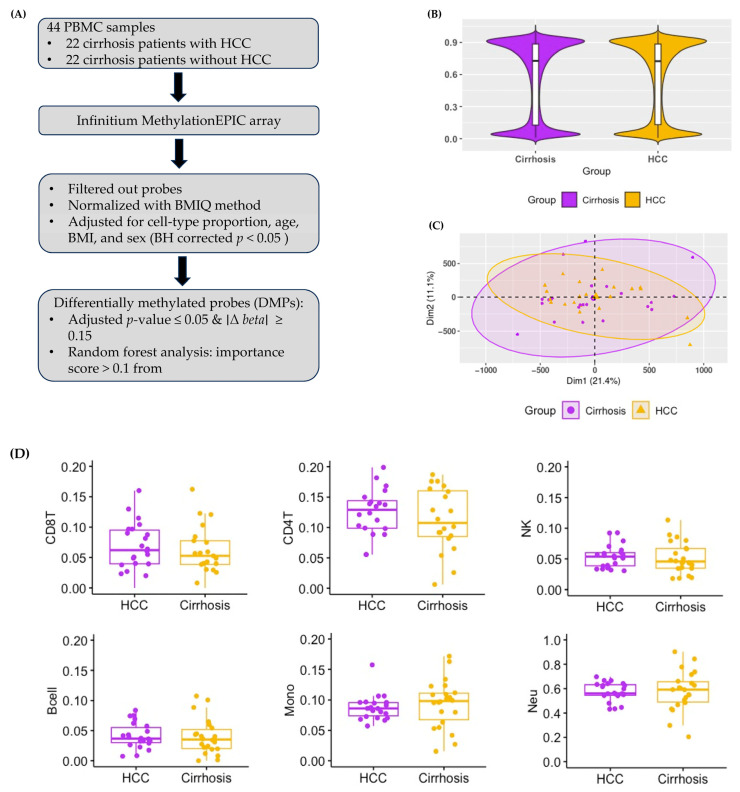

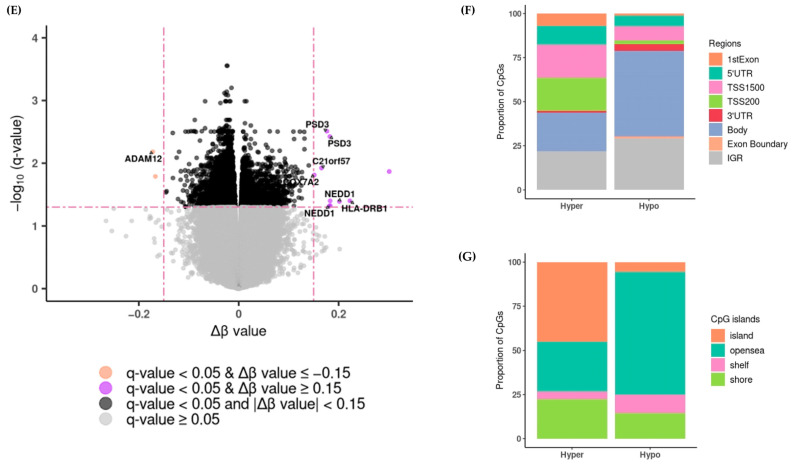
The genome-wide DNA methylation landscape in the buffy coat of cirrhosis patients. (**A**) A flow chart of samples and analysis. (**B**) The average methylation levels of all CpG sites in buffy coat DNA after filtering out the probes targeting the sex chromosomes, probes aligning to multiple locations, and probes with single nucleotide polymorphisms in the probe sequence (median beta values of HCC group = 0.724 and cirrhosis group = 0.727, Wilcoxon rank sum test *p*-value > 0.05). (**C**) The principal component analysis (PCA) was performed using all CpG sites. Each dot represents an individual sample and is shown in different colors: purple dots, cirrhosis patients with HCC cancer; yellow dots, cirrhosis patients without HCC cancer. (**D**) Cell-type proportions of B-cell, CD8+ T cell, CD4+ T cell, natural killer lymphocytes, monocytes, and neutrophils between cirrhosis patients with HCC (the HCC group) and without HCC (the cirrhosis group). (**E**) A volcano plot of differentially methylated probes (DMPs) with an adjusted *p*-value of <0.05 (Benjamini–Hochberg). Purple, significant DMPs with Δ β ≥ 0.15; orange, significant DMPs Δβ ≤ −0.15; and black, DMPs with absolute Δβ ≥ 0.15. (**F**) The distribution of DMPs by the location relative to their genomic regions: gene body, 3′-UTR (3′-untranslated region), intergenic region, exon boundary, TSS1500 (within 1500 bp upstream of the transcription start site), TSS200 (within 200 bp upstream of the transcription start site), 5′-UTR (5′-untranslated region), and 1stExon. (**G**) The distribution of DMPs according to distance from CpG island: CpG island (a short DNA sequence that contains a high cytosine and guanine nucleotides, as well as a high number of CpG dinucleotides compared to the rest of the genome), open sea (isolated CpG sites that are >4 kb from CpG island), shore (region 0–2 kb from CpG island), and shelf (regions 2–4 kb from CpG island).

Figure 1A shows an overview of the study design. After filtering out the low-quality probes, we identified 742,949 methylated CpG sites. The median methylation levels across all CpG sites in buffy coats for the HCC and cirrhosis groups were 0.724 and 0.727, respectively (Figure 1B). Figure 1C shows the principal component analysis (PCA) based on the normalized β values of each CpG site. The median proportions of CD8 T cells, CD4 T cells, natural killer lymphocyte cells, and B-cells in HCC patients were numerically higher than those in cirrhosis patients, but the *p*-values based on the Wilcox rank sum test were >0.05 (Figure 1D). The B-cell had the smallest cell proportion in both the HCC and cirrhosis groups. Thus, the normalized β values were corrected for the cell types excluding B-cells and significant covariates (plate, sex, and BMI) using the linear regression method. 

We identified 8802 DMPs, which is an absolute difference of β values between the HCC and cirrhosis groups of ≥0.15 in a total of 6410 genes (Figure 1E). Among these, hyper-DMPs were 2342 in 1828 genes, and hypo-DMPs were 6460 in 4582 genes in patients with HCC (Supplementary Table S1). The classification of DMPs according to their location relative to genomic regions revealed that the largest proportion of hypo-DMPs was located in the gene bodies and intergenic regions (IGRs) compared to hyper-DMPs (Figure 1F). Most hyper-DMPs were located in the promoter regions that regulate gene transcription. Figure 1G shows that the proportion of DMPs in open sea was the largest in hypo-DMPs while the CpG island was the largest in hyper-DMPs (Figure 1G). 

**Figure 2:**
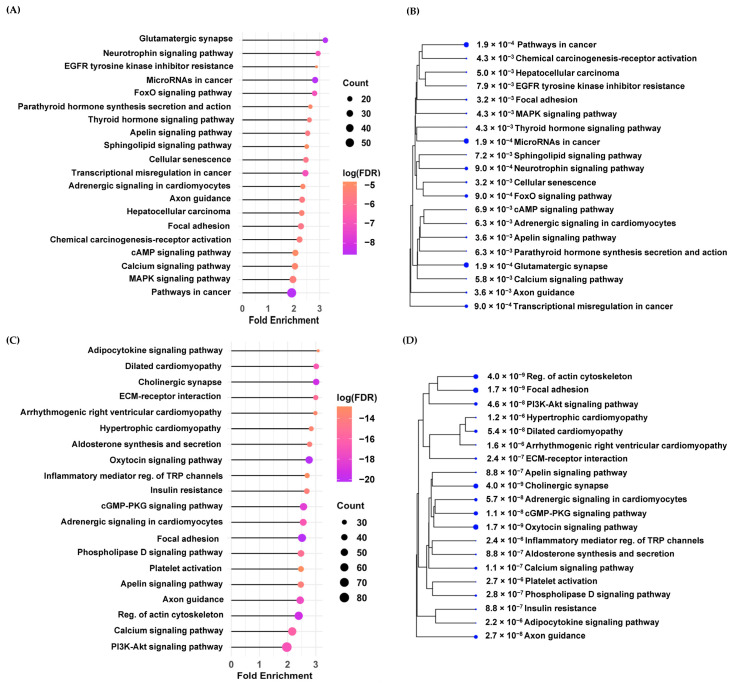
KEGG (Kyoto Encyclopedia of Genes and Genomes) enrichment analysis of the genes corresponding to differentially methylated probes (DMPs). (**A**) The top 20 potential pathways associated with genes corresponding to hyper-DMPs between HCC cases and cirrhosis controls according to the false discovery rates (FDRs) and fold enrichment values. (**B**) A hierarchical clustering tree that grouped the top 20 pathways related to the hyper-DMPs based on how many genes the pathways shared. The number and dots indicate FDRs. Bigger dots indicate more statistical significance. (**C**) The top 20 potential pathways associated with genes corresponding to hypo-DMPs between HCC cases and cirrhosis controls according to the false discovery rates (FDRs) and fold enrichment values. (**D**) A hierarchical clustering tree that grouped the top 20 pathways related to the hypo-DMPs based on how many genes the pathways shared.

Figure 2 shows the top 20 potential KEGG pathways associated with genes corresponding to DMPs between HCC cases and cirrhosis controls according to the false discovery rates (FDRs) and fold enrichment values. Genes corresponding to the hyper-DMPs were mainly enriched in cancer-related pathways, including EGFR tyrosine kinase inhibitor resistance, FoxO signaling, HCC, chemical carcinogenesis-receptor activation and cAMP signaling pathways, and pathways associated with the dysregulation of gene expression, such as the neurotrophin signaling pathway, the apelin signaling pathway, and the mitogen-activated protein kinase (MAPK) signaling pathway (Figure 2A,C, Supplementary Table S2). Genes corresponding to the hypo-DMPs were also enriched in the pathways related to cancer progress (oxytocin signaling, GMP-protein kinase G (PKG) signaling, apelin signaling, and PI3K-Akt signaling). We identified that hypo-DMP-related genes were enriched in pathways related to the immune response (i.e., the adipocytokine signaling pathway, the inflammatory mediator regulation of TRP channel pathway, the platelet activation pathway, and the calcium signaling pathway) (Figure 2C, Supplementary Table S2). Figure 2B,D show hierarchical clustering trees that grouped the top 20 pathways related to the DMPs based on how many genes the pathways shared. 

### 3.3. Identification of Significant DNA Markers

We identified five DMPs in four genes (*PSD3*, *GRB10*, *RGL1*, and *BLCAP*) with an importance score > 0.1. Twelve significant DMPs were screened according to the β values-based threshold (absolute Δβ ≥ 0.15), including ten significantly hyper-DMPs and two significantly hypo-DMPs, corresponding to eight known genes (*PSD3*, *ADAM12*, *C21orf57*, *COX7A2*, *HLA-DRB1*, and *NEDD1*) (Supplementary Table S2). Two out of the 10 significant hyper-DMPs (cg25716013/*COX7A2* and cg00481382/*NEDD1*) were within the TSS1500, and 5′-UTR, respectively (Supplementary Table S1). cg24595678 in *PSD3* was detected as a significant DMP based on the importance score and the Δβ values. 

### 3.4. Association of the Selected DMPs with HCC Prognosis from Cirrhosis

**Figure 3:**
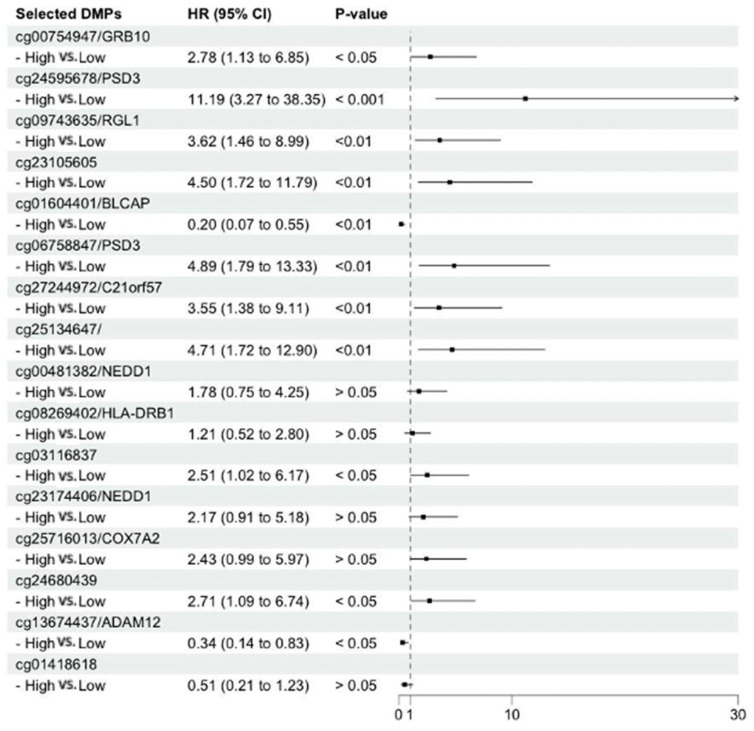
Forest plot of the association of β values of the 16 DMPs and covariates with the HCC prognosis of cirrhosis patients. The hazard ratio (HR) and 95% confidence interval (CI) were derived based on univariate Cox regression models. The selected DMPs were divided into the high group (>median β value of the individual DMP) and the low group (<median β value of the individual DMP).

Figure 3 shows the hazard ratios (HRs) for HCC compared to cirrhosis by the β value (reference: lower than the median) of the 16 DMPs. The HRs were statistically significant for 11 DMPs (nine hyper-DMPs and two hypo-DMPs) (Figure 3, and Supplementary Table S1; four of these DMPs are newly included CpG sites in the 850K array (cg09743635/*RGL1*, cg13674437/*ADAM12*, cg06758847/*PSD3*, cg24595678/*PSD3*).

### 3.5. DNA Methylation Landscape of Buffy Coat Excluding the Hepatitis C Virus (HCV) Active Cases at Baseline

We conducted a sensitivity analysis by excluding the six patients with HCV-active cirrhosis etiology (two from the HCC group and four from the cirrhosis group). We identified 740,917 methylated CpG sites, and the median methylation levels of all CpG sites for HCC and cirrhosis groups were 0.725 and 0.727, respectively. The proportions of neutrophils remained the highest, while B-cells were the lowest. 

After adjusting the β values for CD8+ T cells, CD4+ T cells, natural killer lymphocytes, monocytes, neutrophils, and significant covariates (plate, sex, and BMI) by linear regression method, we identified 11,680 DMPs in a total of 8614 genes, among which, 3173 CpG sites were hypermethylated (hyper-DMPs) in 2401 genes, and 8507 CpG sites were hypomethylated (hypo-DMPs) in 6213 genes in patients with HCC (Supplementary Table S2). A volcano plot was generated to visualize the DMPs (Supplementary Figure S1A). The proportion of DMPs according to their location relative to genomic regions and CpG island remained the same as previously (Supplementary Figure S1B,C). 

Twenty-four DMPs were screened according to the threshold absolute Δβ ≥ 0.15, including 11 significantly hyper-DMPs and 13 significantly hypo-DMPs, corresponding to 15 known genes (*ADAM12*, *LTB4R2*, *STX2*, *PRR12*, *KLHL31*, *ELMOD1*, *UBAP2L*, *LCLAT1*, *LCE3C*, *LCLAT1*, and *MTUS1*). We identify two DMPs (cg06758847 and cg24595678) in *PSD3* based on the importance score from the RF model (Supplementary Table S3). The univariate Cox proportional hazard regression analysis was statistically significant for 10 DMPs in six genes (*ADAM12*, *LTB4R2, STX2, PSD3*, *PRR12*, and *KLHL31* (Supplementary Table S2). Three DMPs of those (cg13674437/*ADAM12*, cg06758847/*PSD3*, and cg24595678/*PSD3)* showed statistically significant association in the primary and secondary analysis (Supplementary Tables S1 and S3). 

## 4. Discussion

We used prospectively collected buffy coat samples from a large cohort to examine DNA methylation patterns in baseline buffy coats of patients with cirrhosis. We identified specific methylated sites associated with the transition from cirrhosis to HCC within the next four years. Most of the DMPs associated with HCC progression from cirrhosis were hypomethylated DMPs (80%). Previous studies reported similar observations on the tissue or blood methylation patterns of HCC compared to those of cirrhosis [11,42]. The methylation level of the three DMPs (cg13674437/*ADAM12*: HR (95% CI) = 0.34 (0.14–0.83), cg06758847/*PSD3*: HR (95% CI) = 4.89 (1.79–13.33), and cg24595678/*PSD3*: HR (95% CI): 11.19 (3.27–38.35) showed a strong association with HCC progression from cirrhosis regardless HCV infection status at enrollment. To our knowledge, this is the first comprehensive epigenome-wide study using Illumina Infinium MethylationEPIC BeadChips with prospectively collected DNA samples from cirrhosis patients.

In our study, the methylation level of cg13674437/*ADAM12* (a disintegrin and metalloproteinase) was lower, and cg06758847/*PSD3* (Pleckstrin and Sec7 Domain Containing), and cg24595678/*PSD3* were higher in cirrhosis patients who subsequently developed HCC than those who remained cancer-free. Gene expression studies of HCC reported that the *ADAM12* gene is highly expressed in HCC tissues and associated with poor prognosis, suggesting a potential biomarker for liver cancer diagnosis [43]. The downregulated *PSD3* by the short interfering of RNA reduces intracellular lipid content, increasing the risk of fatty liver, inflammation, and fibrosis [44]. It has been shown to inhibit HCC cell migration and invasion in an in vitro study [45]. A large Taiwanese prospective cohort study with 16 years of follow-up, in which 237 HCC cases developed, reported that the overall predictive accuracy of hypermethylated genes (*CDKN2A*, *RASSF1A*, *STEAP4*, *TBX2*, *VIM*, and *ZNF154*) in HCC tumors compared to adjacent tissues was 89% with 84% sensitivity and 94% specificity [25]. The same group also profiled prediagnostic plasma DNA methylation and identified hypermethylated *TBX2,* highly associated with increased HCC risk (OR = 3.2, 95% confidence interval: 1.8–6.0), adjusting for age, HBV surface antigen (HBsAg) status, and anti-HCV status [46]. Slowly et al. conducted a large prospective study of Indigenous Americans and performed epigenetic genome-wide analysis using blood from cancer-free individuals. The average flow-up duration for individuals who remained cancer-free was 25.1 years, and for some cases, 11 years. Although the study identified nine DNA methylation alterations that were associated with liver cancer mortality, none of the CpGs were statistically significantly methylated in our study [26]. These different findings between our study and the other prospective studies could be from different types of sources (plasma vs. buffy coat), controls (HCC free, cirrhosis), and the differences in sample sizes and the number of probes covered in each study. Further studies of large prospective cohorts of cirrhosis patients are necessary to validate our findings of discovered epigenomic alterations and to understand the underlying mechanisms of HCC development from cirrhosis.

DNA methylation is more stable than other classes of biomarkers, such as RNA or protein-based markers. Epigenetic alterations in blood represent immune responses; however, they also reflect molecular and cellular changes influenced by genetics, diet, lifestyle, stressors, and environment. When these exposures are repeated over time, epigenetic alterations can lead to changes in the expression of oncogenes and tumor suppressor genes, resulting in loss or gain of gene function [47,48]. Our KEGG enrichment analysis showed that most of the genes corresponding to the DMPs (both hyper and hypo) were mainly enriched in the cancer pathway, pathways associated with the dysregulation of gene expression, and pathways related to cancer progress. Hypo-DMP-related genes were enriched in pathways directly related to the immune response (i.e., the adipocytokine signaling pathway, inflammatory mediator regulation of the TRP channel pathway, the platelet activation pathway, and the calcium signaling pathway). These findings also follow a prospective study exploring the epigenetic differences in peripheral blood, which revealed that differentially methylated CpGs between invasive breast cancer cases and controls were statistically significantly enriched for breast cancer-related pathways and cellular immune responses [49].

Together, our findings support that early methylation alterations arise near cancer and immune response-related genes, and these alterations are associated with cancer prognosis in the susceptible population. Our study used buffy coats obtained from pre-diagnostic samples. We excluded samples from patients who were followed up for less than one year and followed up every six months to eliminate time-related bias. Therefore, the epigenetic alterations observed in our study are more likely influenced by environmental factors rather than detecting cell-free DNA or circulating tumor DNA.

## 5. Conclusions

We identified buffy coat DNA methylation biomarkers that may be associated with HCC risk in cirrhosis patients using pre-diagnostic samples and compared these to patients who did not develop HCC. A further study with a large prospective cohort is required to validate whether our findings can be leveraged to develop biomarkers for the screening, surveillance, and risk stratification of HCC in patients with cirrhosis.

## Figures and Tables

**Table 1:** Clinical and pathological characteristics of patients at baseline.

	Cirrhosis with Future HCC (n = 22)	Cirrhosis Without HCC (n = 22)
Sex		
Female	6 (27.3)	6 (27.3)
Male	16 (72.7)	16 (72.7)
Age: Median (Std Dev)	66 (6.7)	66 (6.7)
Drinking Alcohol		
Never	3 (13.6)	7 (31.8)
Current	3 (13.6)	5 (22.7)
Past	16 (72.7)	10 (45.5)
Cigarette smoking		
Never	5 (22.7)	8 (36.4)
Current	4 (18.2)	6 (27.3)
Past	13 (59.1)	8 (36.4)
BMI: Median (Std Dev)	30.8 (7.1)	29.2 (6.6)
Etiology		
NAFLD	4 (18.2)	7 (31.8)
HCV active	2 (9.1)	4 (18.2)
HCV cured	11 (50)	7 (31.8)
Alcohol-related	3 (13.6)	4 (18.2)
Others	2 (9.1)	0
Hypertension		
No	11 (50)	13 (59.1)
Yes	11 (50)	9 (40.9)

## Data Availability

All analyses were performed in RStudio version 4.3.2 for data wrangling and visualization. Data are available within the article or its Supplementary Materials. Further information is available on request from the authors.

## Supplementary Materials

The following supporting information can be downloaded at https://www.mdpi.com/article/10.3390/cancers17020266/s1, Figure S1: Genome-wide DNA methylation landscape in buffy coats of cirrhosis patients who were not diagnosed with HCV active at baseline; Table S1: Features of differentially methylated probes (DMPs) among cirrhosis patients with HCC compared to patients without HCC; Table S2: KEGG (Kyoto Encyclopedia of Genes and Genomes enrichment analysis of the genes corresponding to the differentially methylated probes (DMPs) with adjusted *p*-value < 0.05 Table S3: Features of differentially methylated probes (DMPs) among cirrhosis patients with HCC compared to patients without HCC after excluding all HCV-active patients from the analysis.
