# Supplementary material for: DNA Methylation Profile in Buffy Coat Identifies Methylation Differences Between Cirrhosis with and Without Hepatocellular Carcinoma
Source: Cancers (Basel). 2025 Jan 15;17(2):266. doi: 10.3390/cancers17020266 (PMC11763440; doi:10.3390/cancers17020266)
Supplement: Supplementary file 1 [file cancers-17-00266-s001.zip › Supplementary Figures.pdf]

Figure legends

Supplementary Figure S1. Genome-wide DNA methylation landscape in buffy coats of cirrhosis patients who were not diagnosed with HCV active. A) Volcano plot of differentially methylated probes (DMPs) with adjusted p-value < 0.05 (Benjamin–Hochberg). Purple, significant DMPs with $\Delta\beta \geq 0.15$; Orange, significant DMPs $\Delta\beta \leq -0.15$, Grey, DMPs with $|\Delta\beta \geq 0.15|$. **B)** Distribution of DMPs by the location relative to their genomic regions: gene body, 3′-UTR (3′-untranslated region), intergenic region, exon boundary (ExonBnd), TSS1500 (within 1,500 bp upstream of the transcription start site), TSS200 (within 200 bp upstream of the transcription start site), 5′-UTR (5′-untranslated region), and 1stExon. **C)** Distribution of DMPs according to CpG islands: CpG island (a short DNA sequence that contains a high cytosine and guanine nucleotides, as well as high number of CpG dinucleotides compared to the rest of the genome), opensea (isolated CpG sites that are > 4kb from CpG island), shore (region 0-2kb from CpG island), and shelf (regions 2-4 kb from CpG island). **D)** KEGG (Kyoto Encyclopedia of Genes and Genomes) Enrichment analysis of genes Functional analysis of differentially methylated probes (DMPs). The potential pathways associated with genes corresponding to hyper-DMPs between HCC cases and cirrhosis controls. **E)** The potential pathways associated with genes corresponding to hypo-DMPs between HCC cases and cirrhosis controls. **F)** The potential pathways from KEEG databases associated with genes corresponding to hypo-DMPs between HCC cases and cirrhosis controls. **G)** A hierarchical clustering tree summarizes the correlation among significant pathways related to the hypo-DMPs. **H)** A histogram of significant DMPs from the analysis including all patients and the sensitivity analysis without HCV active patients.

A)

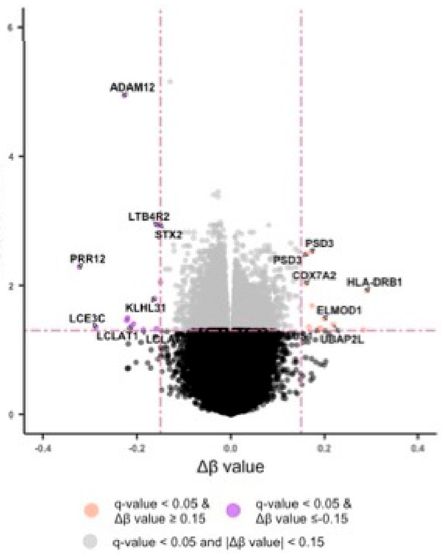
-log10 (q-value)
Δβ value
ADAM12
LTB4R2
STX2
PRR12
KLHL31
LCE3C
LCLAT1
PSD3
PSD3
COX7A2
HLA-DRB1
ELMOD1
UBAP2L
q-value < 0.05 & Δβ value ≥ 0.15
q-value < 0.05 & Δβ value ≤-0.15
q-value < 0.05 and |Δβ value| < 0.15

B)

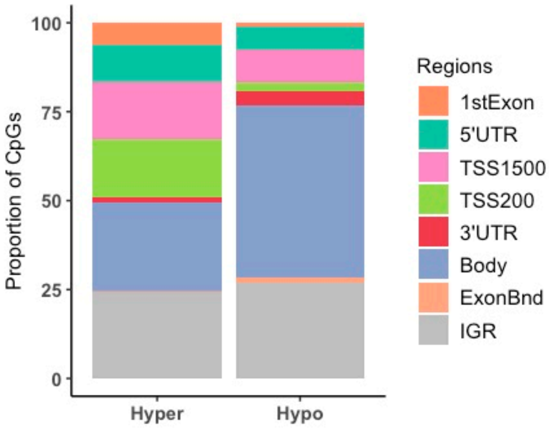
Proportion of CpGs
100
75
50
25
0
Hyper
Hypo
Regions
1stExon
5'UTR
TSS1500
TSS200
3'UTR
Body
ExonBnd
IGR

C)

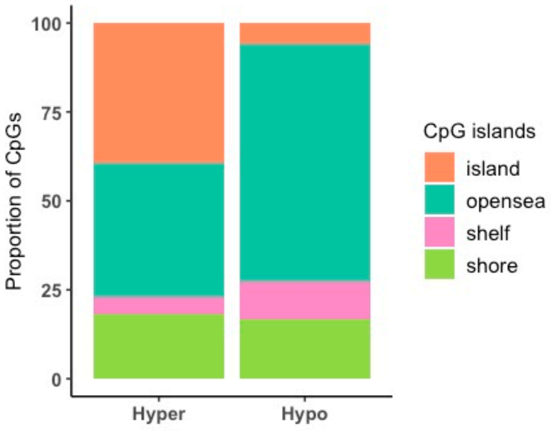
Proportion of CpGs
100
75
50
25
0
Hyper
Hypo
CpG islands
island
opensea
shelf
shore

D)

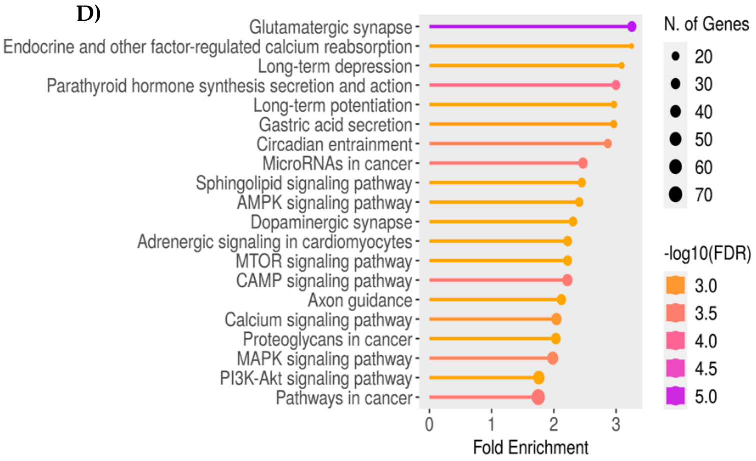

E)

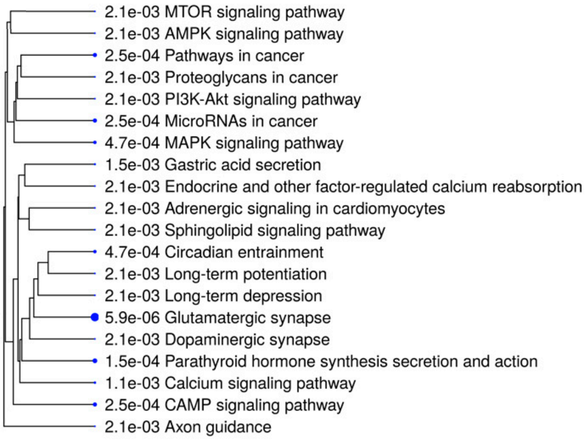

F)

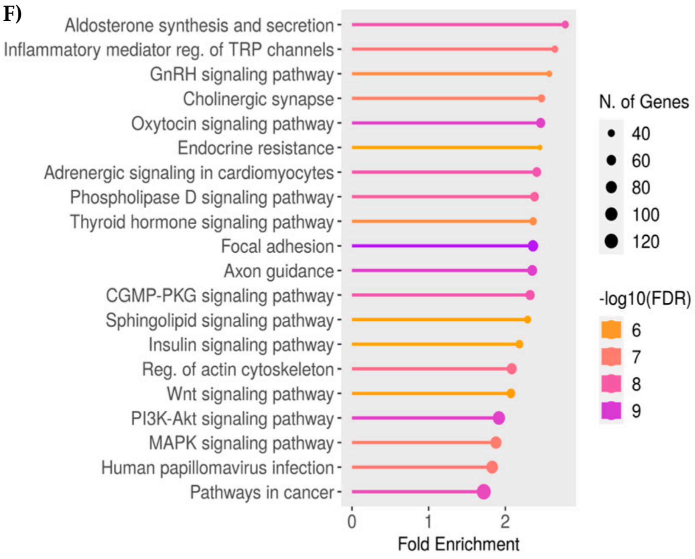

G)

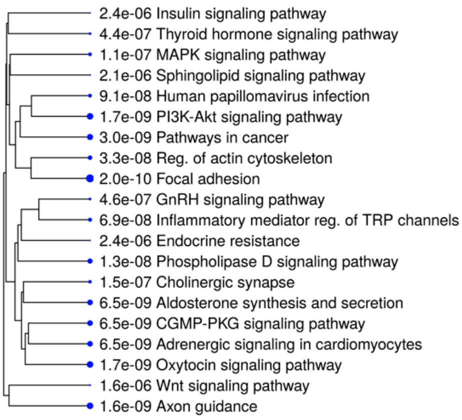

H)

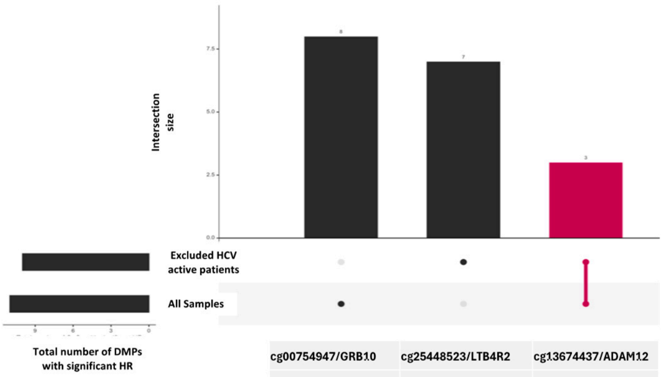

cg00754947/GRB10	cg25448523/LTB4R2	cg13674437/ADAM12
cg09743635/RGL1	cg10832093/STX2	cg06758847/PSD3
cg23105605	cg06378142/PRR12	cg24595678/PSD3
cg01604401/BLCAP	cg04316580/KLHL31	
cg27244972/ C21orf57	cg25374269	
cg25134647	cg15443223	
cg03116837	cg26146732	
cg24680439		

Supplementary Figure S1. Genome-wide DNA methylation landscape in buffy coats of cirrhosis patients who were not diagnosed with HCV active. A) Volcano plot of differentially methylated probes (DMPs) with adjusted p-value < 0.05 (Benjamin–Hochberg). Purple, significant DMPs with $\Delta\beta \geq 0.15$; Orange, significant DMPs $\Delta\beta \leq -0.15$, Grey, DMPs with $|\Delta\beta \geq 0.15|$. **B)** Distribution of DMPs by the location relative to their genomic regions: gene body, 3′-UTR (3′-untranslated region), intergenic region, exon boundary (ExonBnd), TSS1500 (within 1,500 bp upstream of the transcription start site), TSS200 (within 200 bp upstream of the transcription start site), 5′-UTR (5′-untranslated region), and 1stExon. **C)** Distribution of DMPs according to CpG islands: CpG island (a short DNA sequence that contains a high cytosine and guanine nucleotides, as well as high number of CpG dinucleotides compared to the rest of the genome), opensea (isolated CpG sites that are > 4kb from CpG island), shore (region 0-2kb from CpG island), and shelf (regions 2-4 kb from CpG island). **D)** KEGG (Kyoto Encyclopedia of Genes and Genomes) Enrichment analysis of genes Functional analysis of differentially methylated probes (DMPs). The potential pathways associated with genes corresponding to hyper-DMPs between HCC cases and cirrhosis controls. **E)** The potential pathways associated with genes corresponding to hypo-DMPs between HCC cases and cirrhosis controls. **F)** The potential pathways from KEEG databases associated with genes corresponding to hypo-DMPs between HCC cases and cirrhosis controls. **G)** A hierarchical clustering tree summarizes the correlation among significant pathways related to the hypo-DMPs. **H)** A histogram of significant DMPs from the analysis including all patients and the sensitivity analysis without HCV active patients.
